# Human Skeletal Stem Cell Response to Multiscale Topography Induced by Large Area Electron Beam Irradiation Surface Treatment

**DOI:** 10.3389/fbioe.2018.00091

**Published:** 2018-07-24

**Authors:** Vitali Goriainov, Richard B. Cook, James W. Murray, John C. Walker, Douglas G. Dunlop, Adam T. Clare, Richard O. C. Oreffo

**Affiliations:** ^1^Centre for Human Development, Stem Cells and Regeneration, University of Southampton, Southampton, United Kingdom; ^2^Engineering and the Environment, University of Southampton, Southampton, United Kingdom; ^3^Manufacturing Engineering, University of Nottingham, Nottingham, United Kingdom

**Keywords:** nanotopography, surface roughness, skeletal stem cells, osteoinduction, large area electron beam melting

## Abstract

The healthcare socio-economic environment is irreversibly changing as a consequence of an increasing aging population, consequent functional impairment, and patient quality of life expectations. The increasing complexity of ensuing clinical scenarios compels a critical search for novel musculoskeletal regenerative and replacement strategies. While joint arthroplasty is a highly effective treatment for arthritis and osteoporosis, further innovation and refinement of uncemented implants are essential in order to improve implant integration and reduce implant revision rate. This is critical given financial restraints and the drive to improve cost-effectiveness and quality of life outcomes. Multi-scale modulation of implant surfaces, offers an innovative approach to enhancement in implant performance. In the current study, we have examined the potential of large area electron beam melting to alter the surface nanotopography in titanium alloy (Ti6Al4V). We evaluated the in vitro osteogenic response of human skeletal stem cells to the resultant nanotopography, providing evidence of the relationship between the biological response, particularly Collagen type I and Osteocalcin gene activation, and surface nanoroughness. The current studies demonstrate osteogenic gene induction and morphological cell changes to be significantly enhanced on a topography Ra of ~40 nm with clinical implications therein for implant surface treatment and generation.

## Introduction

The global healthcare socio-economic landscape is undergoing irreversible changes as a consequence of an increasing aging demographic. Although the age-specific incidence of physical disability has declined in a number of countries (Jacobzone and Robine, 2001), the unprecedented pace of population aging (UnitedNations, 2013) has resulted in a rise in the prevalence of age-related chronic diseases (i.e., arthritis, osteoporosis) and resultant functional impairment. Together with rising patient expectations of quality of life, these factors have led to a shift toward the development of novel musculoskeletal regenerative and replacement strategies.

Joint arthroplasty is highly effective in the treatment of arthritis and osteoporosis (Chang et al., 1996; Learmonth et al., 2007; Wright and Porteous, 2015). Indeed, compared to cemented counterparts, excellent clinical results and cost-effectiveness have been attributed to the application of uncemented acetabular components (Pennington et al., 2013a,b). However, the UK National Joint Registry has detailed inferior long-term performance of uncemented femoral stems (NJR, 2015). This has called to question the current trend in the dominance of uncemented fixation (Manktelow and Bloch, 2015; Wright and Porteous, 2015). Nevertheless, uncemented femoral components have demonstrated superior survival in younger, more active patient cohorts (Wyatt et al., 2014). The common modes of uncemented hip failure are typically related to the surgical technique employed amongst other factors, emphasizing the technically unforgiving nature of uncemented fixation and the requirement for enhanced implantation expertise by the clinician involved (MacInnes et al., 2012).

Critically, uncemented implant survival in a patient, depends on the transferred load density (P = F/A), which can be minimized through the enhancement of bone/implant contact. To date, the most commonly observed mode of implant failure, accounting for 24.3 and 29.8% of all failures in total hip and total knee arthroplasties respectively, is aseptic loosening (Khan et al., 2016; NJR, 2016). Aseptic loosening is triggered by bearing surface wear and release of particulates or tribological debris. De Maeztu and colleagues reported that only 33–62% of bone-implant contact is achieved by modern titanium implants subject to commercially available surface treatments after 3–6 months (De Maeztu et al., 2008). Attainment of 100% bone-implant contact area would yield a reduced interface load density and elimination of the wear debris entry into the interface plane, thus precluding the inflammatory and osteolytic processes resulting in aseptic loosening. These observations demonstrate the importance of research efforts focused on enhancing the establishment of a robust and durable bone-implant interface.

Following early uncemented implant failures, the first regular use of total hip replacement implants, in the late 1950's, employed a cemented fixation method (Charnley, 1961; McKee and Watson-Farrar, 1966). However, loosening of these cemented implants led to further research to establish improved outcomes through development of cementless techniques. The refinements in uncemented components included the addition of surface treatments using porous or hydroxyapatite coating to allow bone in- or on-growth. Such an approach resulted in significant improvements in clinical implant survival (McLaughlin and Lee, 2010). The resultant implant surface macro-roughness contributed to mechanical anchorage essential for attainment of primary stability. Further design refinements resulted in the development of porosity essential for vascular formation, proliferation of mesenchymal cells and, ultimately, osteogenesis (Kuboki et al., 1998). Additional improvements included emulation of the natural structure and mechanical properties of the bone lattice using trabecular metal (Cohen, 2002). Surface modification techniques such as plasma spray coating, grit blasting, acid etching, combined sand blasting, and acid etching have been commercially utilized in the surface modification of orthopedic implants (Geetha et al., 2009; Jemat et al., 2015). Future developments will need to focus on enhancement of the implant-bone interface formation through modulation of surface alterations, preferably at a nanotopographical scale recognizable by individual stem cells.

The process of uncemented implant osseointegration is thus essential for attainment of secondary stability and long-term survival. The initial bone-implant interface deficiencies retained following the implantation of press-fit orthopedic implants require native bone infill through the process of osteogenesis. Osteogenesis as observed on the surface of the implant is known as contact osteogenesis (Osborn and Newesely, 1980), and central in implant surface colonization by osteogenic progenitors, synthesis of extracellular bone matrix, and appositional *de novo* bone formation (Davies, 2003). Critically, the ability of nanotopographical scale roughness to stimulate an appropriate osteogenic response from skeletal stem cells (SSCs) through mimicking the nanofeatures naturally present within the bone matrix has been harnessed in recent years to promising effect (Dalby et al., 2007; Sjöström et al., 2009; Lavenus et al., 2012; Zhang et al., 2012).

Large Area pulsed Electron Beam irradiation (LAEB) is a novel method to create metal surface layer modifications (Proskurovsky et al., 1998; Walker et al., 2014). The known benefits of LAEB treatment include: (i) improvement of Ti alloys fatigue characteristics (HCEI, 2015), (ii) enhancement of material strength properties (Proskurovsky et al., 2000), (iii) nano-hardening of the surface and sub-surface with resultant increased resistance to initiation and propagation of cracks (Gao, 2013), and (iv) improvement in the surface corrosion resistance (Walker et al., 2014). However, to date, there has not been any published experimental work exploring the biological potential of LAEB-induced alterations of surface topography.

The current study has examined, *in vitro*, the biological responses induced using LAEB treatment-generated topography on human skeletal stem and progenitor cell populations. The work has focused on the modulation of skeletal stem/progenitor cell function and phenotype in relation to topographical surface characteristics.

## Materials and methods

### Materials, substrate preparation, and characterization

Three different bulk supplies of wrought Ti6Al4V (Ti64) for three *in vitro* studies were purchased from www.ti-shop.com. Samples were prepared by sectioning into cylindrical discs and one surface of each sample was flat lapped using a Kemet 15 precision flat lapping machine utilizing diamond abrasives of 25 to 6 μm grades.

The polished Ti64 surfaces of each sample were irradiated using a Sodick PF32A electron beam-melting machine (Supplementary Figure 1). Nine samples were prepared for each *in vitro* analysis group and LAEB-treated with a range of cathode accelerating voltages for 1, 15, or 25 pulses (Table 1), with constant 11 sec interval between pulses essential for regeneration of beam conditions and cooling of the melted surface.

**Table 1:** Test groups defined by LAEB treatment parameters.

**Group**	**Cathode voltage (kV)**	**Shots**
Untreated (Control)	0	0
15kV1	15	1
15kV15	15	15
15kV25	15	25
25kV1	25	1
25kV15	25	15
25kV25	25	25
35kV1	35	1
35kV15	35	15
35kV25	35	25
40kV15	40	15

*The energy transferred to the surface as a result of electron beam irradiation was directly related to magnitude of voltage and number of pulses*.

The machine consisted of a sample chamber, which was evacuated of air with nitrogen and pressurized to 0.05 Pa with argon gas, which was the medium used for plasma build up. A series of magnetic solenoid coils on the outside of the vacuum chamber produced an electron plasma cloud generating magnetic field, at the maximum intensity of which a 5 kV pulsed voltage was applied to the anode and Penning discharge was initiated. In 50–100 μs, the current of the Penning discharge reached 150–170 A, and a plasma column was formed near the anode. After a further 10–30 μs delay period, an accelerating voltage was applied to the emission cathode, concentrating the electric field of up to 400 kV/cm in a near-cathode ion layer and triggering the explosive emission from a number of cathode electron-emitting spots (dense plasma clouds). The electrons were accelerated and an electron beam was formed in a double-layer, between the cathode plasma and the anode plasma, in which the applied voltage was concentrated. This defocused electron beam pulse was transported through the anode plasma to a collector cathode, where the work piece was placed. The effect of the process on the surface topography depended on the electron accelerating cathode voltage and the number of electron pulses. As a result of the defocused nature of the electron beam, large surface areas can be irradiated with electrons, generating a technique is known as Large Area Electron Beam (LAEB) treatment. Developed for the surface processing of high value engineering components such as mold tool, this technique is well suited to the processing of complex implant surfaces.

Microbiological decontamination of substrates tested was undertaken in PBS/1% antibiotic-antimycotic solution (Life Technologies, Invitrogen, UK) for a minimum of 24 h, before cell seeding in culture plates.

### Human skeletal stem cell (SSC) culture

Adult human bone and osteoprogenitor cells were extracted from the bone marrow samples obtained from haematologically healthy patients undergoing hip replacement surgery with local ethics committee approval (LREC194/99/1), as previously described (Yang et al., 2003). Further enrichment of the SSC fraction from the bone marrow cell population was achieved using the STRO-1 antibody and magnetic sorting, as previously detailed (Howard et al., 2002; Yang et al., 2003; Mirmalek-Sani et al., 2006).

Individual experiments were performed using primary human skeletal stem/progenitor cell enriched cultures from discrete patient donors unadjusted for demographics [3 donors for 3 repeat *in vitro* experiments: 1 female 71 years of age and 2 males 65 and 73 years of age (mean age 69.6 years)].

SSCs were cultured in a basal medium (α-MEM/10% FCS/1% P/S) at 37°C in 5% CO_2_, with medium changes carried out twice weekly. Passage 1 cells were used exclusively in all studies. SSCs were seeded at 220/cm^2^ density either directly onto substrates (test groups) or TCP (control groups) for subsequent 21 day *in vitro* cultures.

### Live/dead cell assay

Cell viability was assessed using CellTracker™ Green (CTG) CMFDA and ethidium homodimer-1 (Life Technologies, Invitrogen, UK). Fifty micrograms of CTG and 5 μg of ethidium homodimer were dissolved in 10 μl of DMSO and subsequently added to the culture medium.

The topography images obtained using white light interferometry and fluorescent cell imaging after 21 days of *in vitro* cultures were superimposed under guidance from intentionally introduced surface defects in predetermined samples in order to correlate the effect of underlying surface topography on cell morphology. Images were subsequently analyzed based on the surface topography mapping scale used in white light interferometry.

### *in vitro* immunocytochemistry

The ability of LAEB treatment to induce bone-specific ECM protein synthesis linked to the osteogenic gene activation was analyzed in Untreated, 25kV25 and 35kV25 groups. After 3 weeks in *in vitro* culture, cells adherent to substrate surfaces were fixed in 4% PFA, blocked and permeabilized in blocking buffer [PBS/5% goat serum in PBS (5 g in 100 ml)/0.3% Triton X-100] for 60 min, and treated overnight in anti-OPN primary antibody raised in rabbit and diluted in Antibody Dilution Buffer (PBS/1% BSA/0.3% Triton X-100) to 1:50 concentration (GeneTex). This was followed by application of goat anti-rabbit IgG (H+L) secondary antibody, Alexa Fluor® 488 conjugate (Life Technologies, Invitrogen, UK) diluted to 1:200 concentration and incubated in the dark for 1 h at room temperature. Counterstaining was performed using DAPI (4′,6-Diamidino-2-Phenylindole, Dihydrochloride) (Life Technologies, Invitrogen, UK). The substrates were mounted on slides followed by imaging. The imaging was undertaken at the same exposure and the fields selected following the examination of an entire substrate were from the center of each sample, representing the staining tendency on each sample.

### Imaging

Image capture was undertaken using a Zeiss Axiovert 200 inverted microscope with an Axiocam MR camera for fluorescent imaging and Axiovert HR camera for white light imaging operated by Zeiss Axiovision software version 4.7.

### Molecular analysis of osteogenic gene expression

Trypsin-EDTA buffer (Sigma-Aldrich) was used to release cells from relevant culture surfaces (8 material replicates) prior to lysis. Total mRNA extraction was accomplished using the Qiagen RNeasy kit in accordance with manufacturer's instructions. The quantities of mRNA obtained ranged between 150 and 300 ng/μL, and the purity as measured by A260/A280 ranged between 1.89 and 2.08 (NanoDrop 1000 spectrophotometer, Thermo Scientific). mRNA samples were treated with DNAse. Seven microliters of RNA was combined with 2 μl 5X VILO reaction mix and 1 μl 10X SuperScript® enzyme for cDNA synthesys and reverse-transcribed using SuperScript first-strand synthesis system (Veriti Thermal Cycler, Applied Biosystems). Twenty microliters of qPCR reaction mixture containing 1 μl of cDNA and 1 μl of each primer was prepared. qPCR using SYBR® Select Master Mix (Life Technologies) was carried out on 7500 Real-Time PCR system (Applied Biosystems) for amplification of β-actin, ALP, Collagen type I, OPN, and OCN genes. β-actin was employed as the reference gene and Collagen type II as a negative control. Ten microliters of reaction volumes with 300 nM primer concentrations were used in all PCR amplifications. Primer sequences (Table 2) were validated by dissociation curve/melt curve analysis and the efficiencies of amplification for the β-actin primers and primers for the bone marker genes of interest were approximately equal. The quantification of PCR amplification data was achieved using the comparative cycle threshold (CT) method and relative transcript levels were expressed as mean ± S.D. The CT values obtained ranged from 15 to 24 cycles. Data were analyzed and plotted using GraphPad Prism 6 for Mac OS X software.

**Table 2:** Primer sequences used for real-time qPCR.

**Gene**	**Primer pairs**	**Amplicon**	**Standard curve slope**	**Efficiency (%)**	
β-Actin	F: 5′ GGC ATC CTC ACC CTG AAG TA 3′	82	−3.41	96.3	NM_001101
	R: 5′ AGG TGT GGT GCC AGA TTT TC 3′				
ALP	F: 5′ GGA ACT CCT GAC CCT TGA CC 3′	86	−3.38	97.6	NM_000478
	R: 5′ TCC TGT TCA GCT CGT ACT GC 3′				
Collagen type IαI	F: 5′ GAG TGC TGT CCC GTC TGC 3′	52	−3.42	96.1	NM_000088
	R: 5′ TTT CTT GGT CGG TGG GTG 3′				
OPN	F: 5′ GTT TCG CAG ACC TGA CAT CC 3′	80	−3.34	99.3	NM_001251830
	R: 5′ CAT TCA ACT CCT CGC TTT CC 3′				
OCN	F: 5′ GGC AGC GAG GTA GTG AAG AG 3′	102	−3.4	96.8	NM_001199662
	R: 5′ CTC ACA CAC CTC CCT CCT 3′				
Collagen type IIαI	F: 5′ CCT GGT CCC CCT GGT CTT GG 3′	58	−3.39	97.2	NM_001844
	R: 5′ CAT CAA ATC CTC CAG CCA TC 3′				

*The efficiency of the primers was calculated using the standard slope plotted with 5 dilutions*.

### Surface topography characterization

Following LAEB treatment, five randomly selected samples from each group were selected for surface topography analysis performed using a Talysurf contact profilometer (Taylor Hobson) with a 2 μm radius tip. The R_a_ of each individual sample was measured five times with the direction of profile measurements systematically changed by rotating samples 20–30 degrees clockwise. To comply with ISO 4288:1998 standards, the traversed length for the roughness measurement was set as 4 mm to provide a total measured length of 3.75 mm, with the profiles filtered using a λ_c_ of 0.25 mm, based on the obtained roughness measures. A third set of samples was analyzed using White Light Interferometry using a 3D optical microscope (Bruker) to validate the findings from contact profilometry.

Following LAEB treatment, the 35kV25 treatment group was observed to undergo significant surface topography R_a_ alterations. Therefore, the same sample, initially untreated and subsequently treated with 35kV25, was further examined using a Zeiss NVision 40 dual beam FEG–SEM microscope operating at 5 kV accelerating voltage and × 5,000 magnification.

### Statistical analysis

Statistical analysis was carried out using Microsoft Excel for Mac 2011 Version 14.6.4 (Microsoft Corporation) and Prism 6 for Mac OS X (GraphPad Software) with data presented as mean ± standard deviations. *In vitro* experiments were repeated three times to ensure validity and reproducibility of the results. Eight independent culture samples were pooled for RT-PCR analysis to minimize the effect of the variation introduced by individual samples. The results were expressed as mean ± standard deviation (SD). Two-way ANOVA tests were used in comparing multiple factors between seven independent test groups. *T-*test was used in comparison for a single factor between two independent groups. The significance level was set at *p* < 0.05.

## Results

### Characterization of surface topography

**Figure 1:**
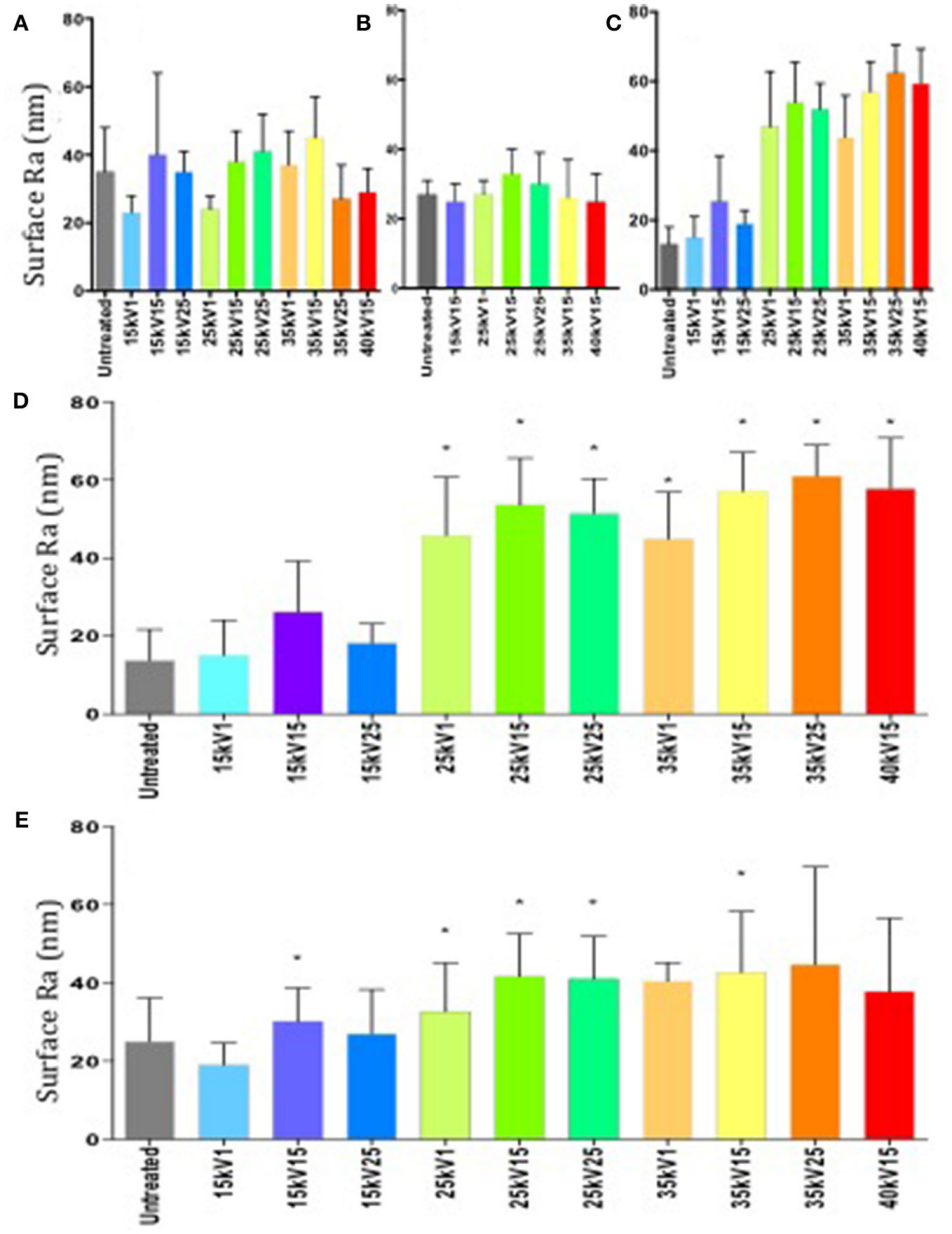
**(A–C)** Surface roughness measurements of R_a_ by contact profilometry. Variation in surface roughness between individual batches of samples was observed (first batch—**A**, second—**B**, third—**C**). Results expressed as mean ± SD, five random samples were analyzed in each group. **(D)** Surface roughness measurements of R_a_ in a third batch of samples determined by white light interferometry. Column statistics employed, results expressed as mean ± SD, five random samples were analyzed in each group, ^*^p < 0.05. **(E)** Combined surface roughness measurements of R_a_ by surface profilometry. Untreated group taken as a negative control and served as the reference point. Column statistics were used, results expressed as mean ± SD, individual measurements from three sample batches were combined, ^*^p < 0.05.

The surface roughness of each individual sample batch was determined following LAEB treatment and observed to exhibit a degree of variation (Figures 1A–C). The findings of surface topography analysis by white light interferometry revealed the R_a_ trend and the scale of the surface topography were comparable to the data obtained from surface contact profilometry undertaken on the same set of samples (Figures 1C,D).

An increase in surface roughness with increasing treatment energy was observed (Figure 1E), although due to the variation between sample batches, not all observations reached statistical significance.

**Figure 2:**
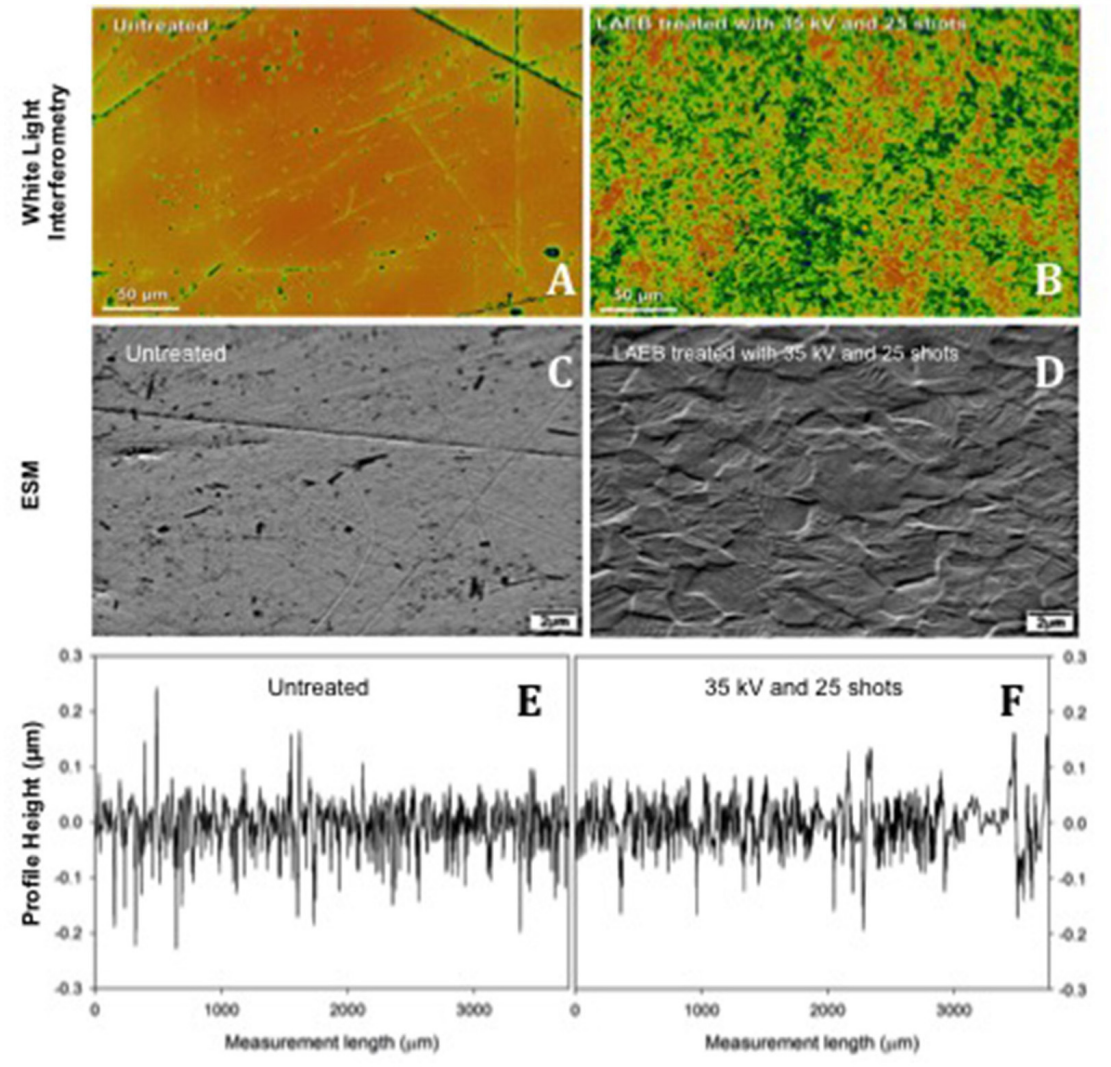
Topography of Untreated **(A,C)** and 35kV25 **(B,D)** samples. Inhomogeneties (surface marks and abrasions) were eliminated following treatment, and an enhanced texture developed. Scale bar 50 μm in **(A,B)** and 2 μm in **(C–F)**. Samples of surface roughness traces from Talysurf contact profilometer for Untreated and 35kV25 surfaces **(E,F)**, respectively.

As a result of repeated surface melting and cooling, the LAEBM treatment resulted in removal of the original surface defects and the subsequent generation of a new surface profile dominated by the presence of nanoscale, grain-like, features (Figure 2).

### Osteogenic marker expression in SSCs following culture on nanosurfaces

**Figure 3:**
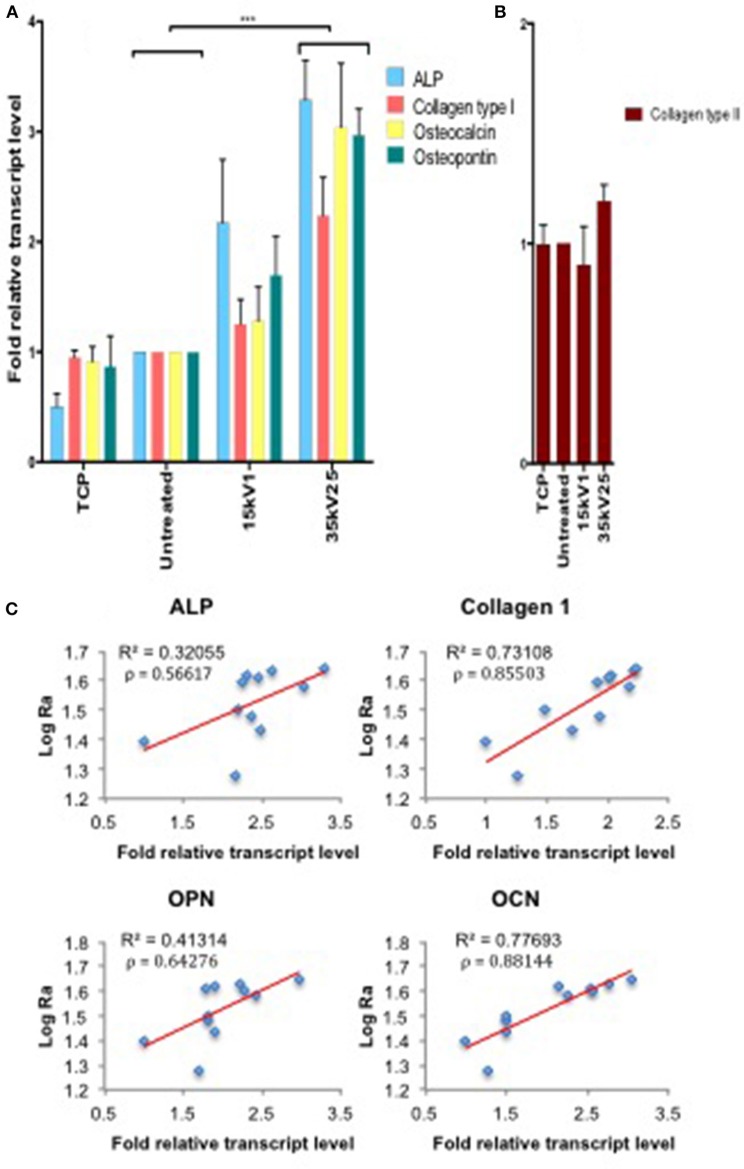
Real time qPCR analysis of osteogenic (ALP, Collagen type I, OPN, and OCN—**A**) and negative control (Collagen type II—**B**) gene expression in STRO 1 SSCs cultured *in vitro* for 21 days. Ti64 Untreated is taken as the negative control. Results expressed as mean ± SD, triplicate samples, individual experiment repeated three times and analyzed using 2-way ANOVA test, ^***^p < 0.001. **(C)** Correlation of individual bone marker gene expression **(A)** vs. Log R_a_, revealing a strong link between surface roughness and Collagen type I and OCN gene induction, as indicated by *R*^2^ and Pearson correlation coefficient (ρ) values.

Osteogenic gene induction for ALP, Collagen type I, OPN, and OCN markers was observed to directly correlate with the surface roughness as measured by R_a_. Furthermore, gene induction was observed to be directly dependent on the LAEB treatment parameters (Figures 3A,B). The highest levels of osteogenic gene induction were found in the experimental sample groups treated with higher voltages in combination with a higher number of pulses (35kV15, 35kV25, and 40kV15; Supplementary Figure 2), with the results reaching statistical significance in comparison to untreated surfaces. The highest stimulation (two- to three-fold enhancement) in bone marker genes was observed in the 35kV25 experimental group, which corresponded with the highest surface R_a_ of 44 nm. A strong correlation between the surface R_a_ and osteogenic potential was demonstrated as evidenced by R^2^ and Pearson's co-efficient (ρ) values. The correlation in surface Ra was notably marked for Collagen 1 and OCN genes with ρ values approaching 1 (Figure 3C).

**Figure 4:**
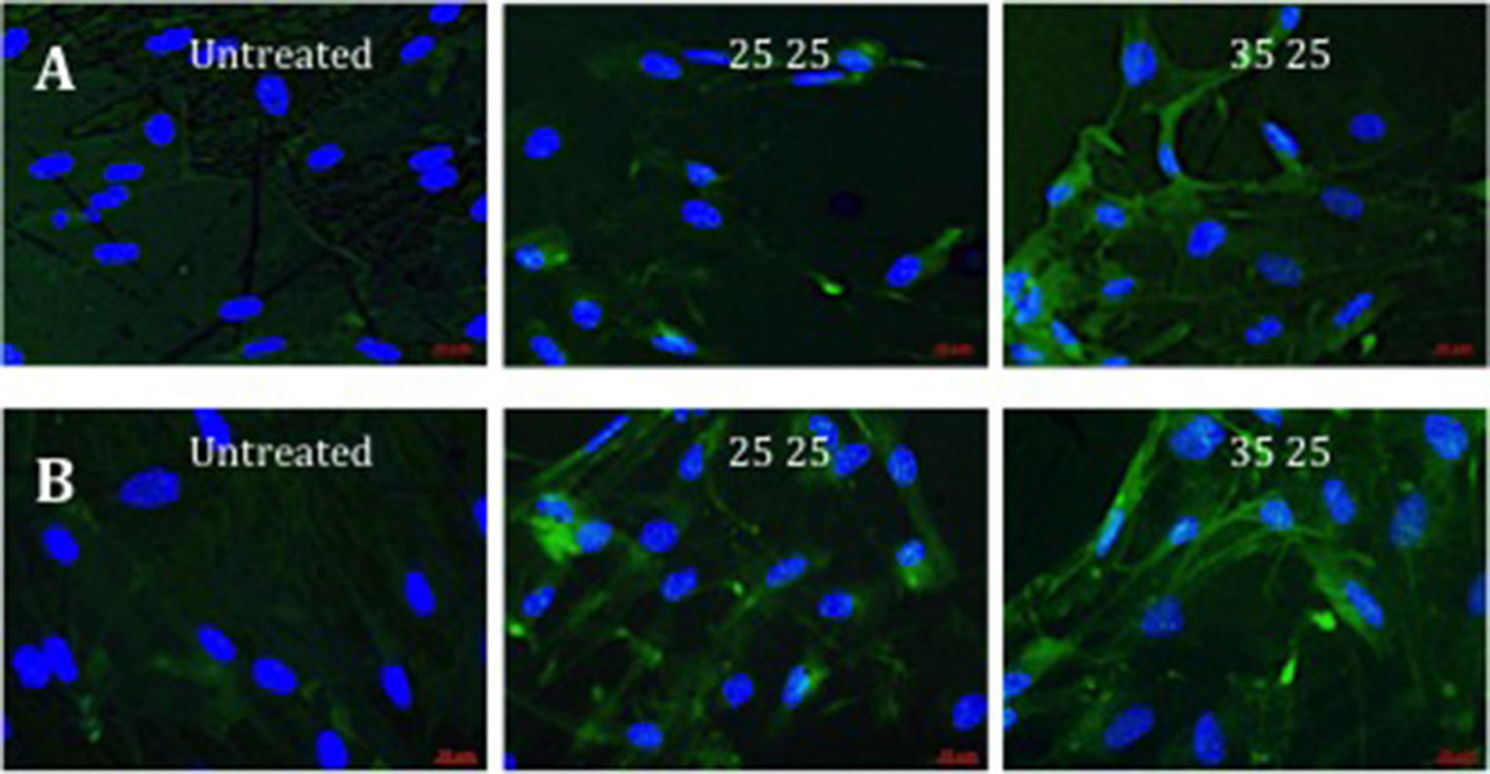
Fluorescence of OPN immunostaining in STRO 1 SSCs cultured *in vitro* on untreated, and 25 and 35kV25 treated Ti64 surfaces. Two repeat sample set images are provided **(A,B)**. Blue fluorescence revealed nuclear counterstain (DAPI) and green fluorescence, OPN (scale bar 20 μm).

Further evidence of the osteoinduction potential of the materials was demonstrated by the enhanced bone-specific extracellular matrix protein expression observed on the treated surfaces with increasing R_a_ (Figure 4). Higher OPN fluorescence and the observed marked spread stellate morphology of the cell populations, characteristic of osteoblasts, noted on 40kV15 surfaces.

### Evaluation of human skeletal stem cell growth and morphology

**Figure 5:**
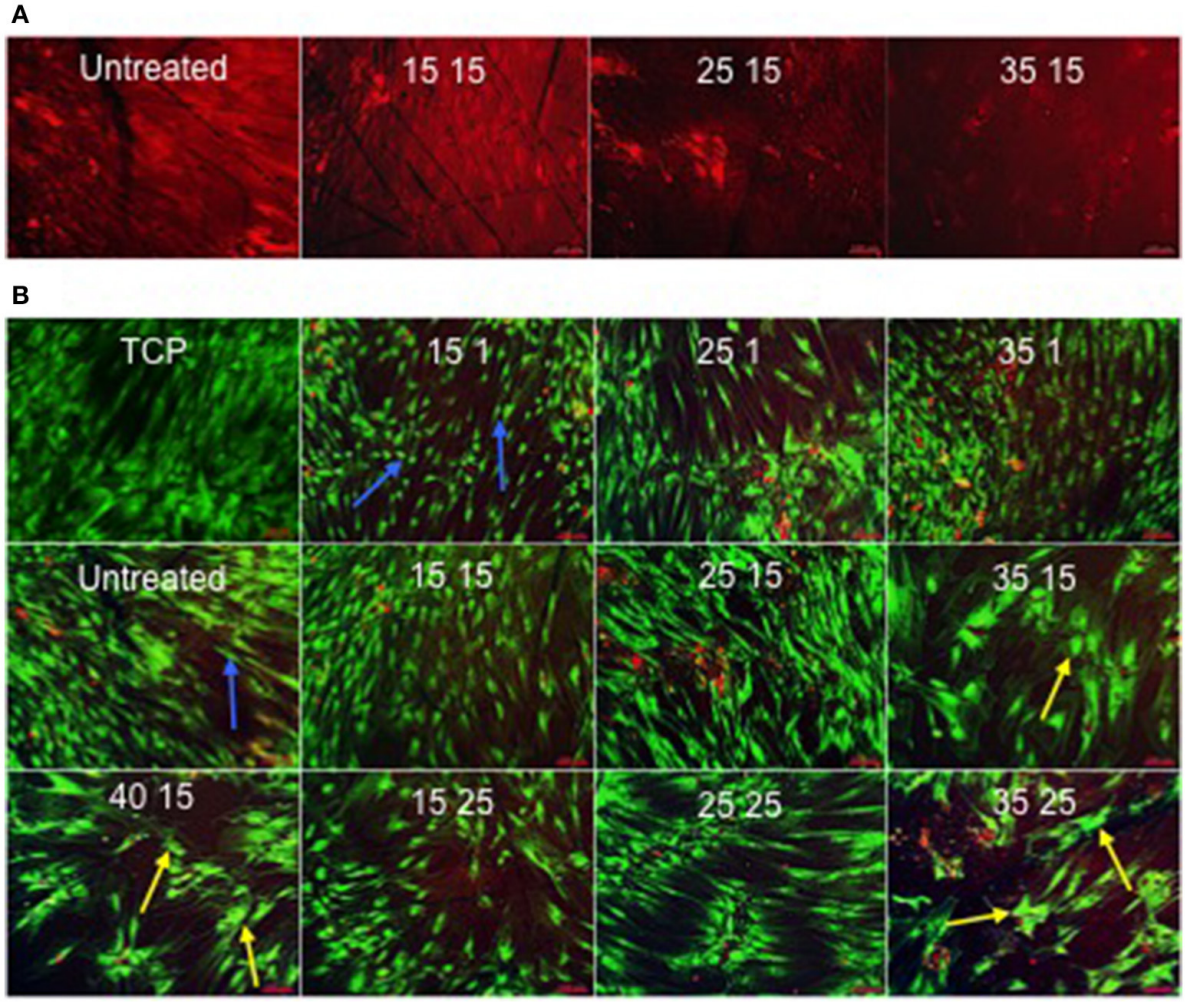
**(A)** Ethidium homodimer-1-induced fluorescence as a consequence of adsorption to the surface. In contrast to 25kV15 and 35kV15 samples, the surfaces of the Untreated and 15kV15 samples contained significant surface abrasions and marks (scale bar 100 μm). **(B)** STRO 1 viability and morphology at 21 days *in vitro* culture on TCP and Untreated control surfaces, and on treated surfaces in basal medium. STRO 1 SSCs on Untreated and 15kV1 surfaces displayed an elongated and fibroblast-like morphology (blue arrows). The flattened, spread and polygonal morphology of STRO 1 SSCs on 25kV15, and on 35kV15, 35kV25, and 40kV15 surfaces, indicative of osteoblast phenotype was noted on surfaces (yellow arrows) (scale bar 100 μm).

The titanium oxide on Ti64 substrate surfaces were noted to adsorb ethidium homodimer-1, creating background fluorescence that defined the topography of the surface (Figure 5A) underlying the cell cultures (Figure 5B).

**Figure 6:**
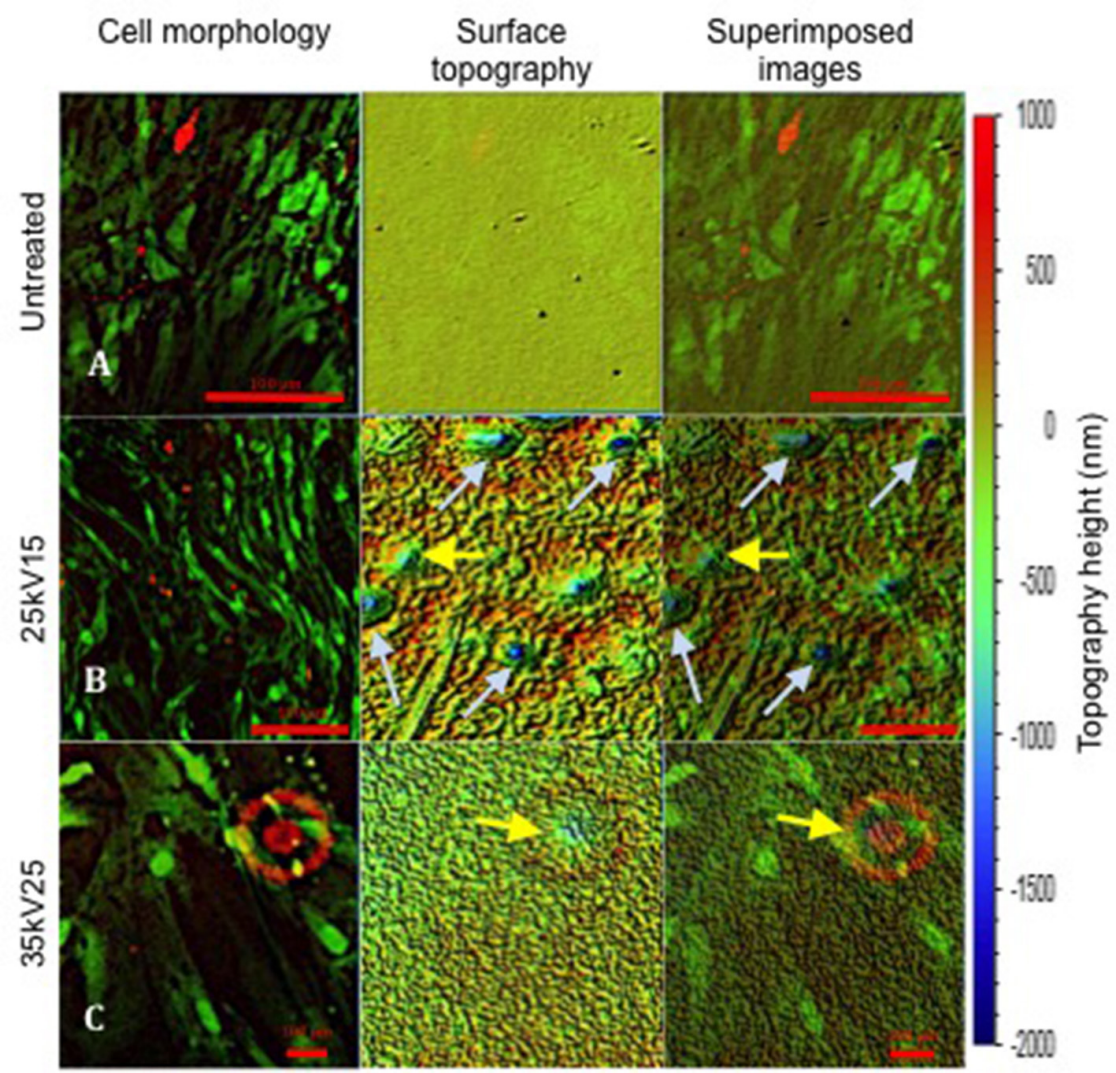
Superimposed images revealing SSCs on the surface of underlying topography at 21 days of *in vitro* culture. The topography change induced following treatment is demonstrated. Yellow arrows indicate topographical pits with depths of ≤ 1,000 nm; light blue arrows indicate pits with depths >1,000 nm. Note the different magnifications in **(A–C)** required to match the initial topography images obtained using white light interferometry (scale bar 100 μm).

Further evaluation of the relationship between osteoblast cell morphology and the underlying topography was achieved by superimposing the images of cell morphology and interferometry-generated surface topography (Figure 6). However, as a consequence of cell layer detachment at the end of *in vitro* cultures, the analysis was only technically possible with untreated, 25kV15 and 35kV25 substrate surfaces. The superimposed field of the untreated surface (Figure 6A) was noted to be flat without any underlying defects. The superimposed field of 25kV15 surface (Figure 6B) presented a number of “craters” produced by localized expulsion of material during the melting process. The 35kV25 surface field (Figure 6C) contained a single shallow crater. The superimposed fields demonstrated the capacity of the osteoblast cells to bridge craters with depths of 500–1,000 nm (yellow arrows) and to line the circumference of the rim of deeper craters of >1,000 nm depth (light blue arrow) without bridging the space.

## Discussion

The current study has demonstrated that LAEB surface treatment using 35kV15, 35kV25, and 40kV15 parameters induced a strong osteogenic differentiation in human SSCs evidenced by morphological changes, and induction of bone marker gene activation and bone matrix protein synthesis. The observed phenomena were related primarily to the surface roughness as measured by R_a_. A strong direct linear regression was demonstrated between the levels of bone marker gene expression and material surface roughness. These findings indicated that within a defined range of nano-scale topography analyzed in this study, higher surface roughness (higher R_a_) resulted in a strong stimulus for osteogenic induction of human SSCs. Additional surface changes potentially induced by the treatment, but not fully characterized under the scope of this research project, may also be at play. The ability of surface topography to stimulate osteogenic differentiation in SSCs has been established (Zhao et al., 2005; Dohan Ehrenfest et al., 2010). However, most of the commonly used industrial surface modifying techniques (i.e., acid etching, grit blasting) generate micro-scale surface topography (Kim et al., 2005; Zhao et al., 2005). Micro-scale topography has been shown to possess the potential for osteoinduction, but not to address the ability of cells to sense and respond to nano-scale physical features naturally present in their niche environment (Olivares-Navarrete et al., 2014).

Xing et al. examined the SSC response to NaOH treatment-induced nanotopography with R_a_ ranging between 13 and 20 nm (Xing et al., 2014) and reported an inverse correlation between surface roughness and hydrophilicity, and a strong direct correlation between hydrophilicity and osteogenic SSC induction. Conversely, Walker and colleagues have previously shown that increasing roughness (R_a_) of LAEB-treated samples resulted in increased hydrophilicity (Walker et al., 2014), indicating a direct correlation between the two surface characterization parameters. This correlation between roughness and hydrophilicity poses difficulty in decoupling their individual effects on cellular response. Nevertheless, to our knowledge, the dynamic trend of SSC responsiveness to osteogenic stimulation by underlying nanotopography with R_a_ ranging from 19 to 44 nm has not, to date, been comprehensively determined highlighting the value of current observations.

The highest R_a_ was observed on 35kV15, 35kV25, and 40kV15-treated Ti64 surfaces, Enhanced attachment of SSCs, likely through the formation of focal adhesion complexes at the grain boundaries (Webster and Ejiofor, 2004), and subsequent cytoskeletal rearrangements and intracellular pathway activation resulting in osteoblast-like morphological changes was observed in these groups. In turn, the cytoskeletal rearrangement and tension, initiated the activation of bone marker genes and synthesis of ECM proteins (Dalby et al., 2014). The importance in relation to cell phenotype and function (including mechanosensor role of the nucleus) within adhesion-cytoskeleton-nucleus mechanotransduction pathway has been firmly established (Wormer et al., 2014). The current studies have not examined the initial steps of osteogenic induction, or subsequent matrix organization and mineralization. Wang et al have shown that cellular function (i.e., Collagen type I synthesis) was dependent on the focal adhesion rearrangements modulating nuclear volume, with the effect being particularly sensitive to the height of nanotopography (Wang et al., 2016). However, Wang et al investigated topography heights ranging from 150 to 560 nm, identifying 150 nm as the optimal height from the range analyzed able to facilitate enhanced focal adhesion rearrangements. With the osteogenic activity observed in our study peaking at R_a_ = 44 nm, it is conceivable that the optimal height of nanotopography required for induction of osteogenic differentiation of SSCs lies at or above this value.

The most striking cell morphological changes were observed on the surfaces with the highest R_a_. Of particular interest, were observations indicating SSCs could bridge the underlying defects ~500 nm deep, however the SSCs appeared to circumvent larger underlying craters. This results could indicate that the topography R_a_ ranging 0.5–1 μm was either beyond the scope sensed by the cells and therefore preferentially avoided, or could be detected and provided further guidance of cell migration around the rim resulting in circumferential attachment. Wang et al. previously reported human lung fibroblasts failed to conform to the underlying topography, but could bridge gaps with heights of 560 nm (Wang et al., 2016), possibly due to cell membrane elasticity preventing radical bending required to reach the bottom of topographical pit (Ohara and Buck, 1979).

The variation in surface topography scale between the three sets of samples was likely due to minor variations in Ti6Al4V composition and levels of impurities between the three wrought Ti6Al4V bulk supplies. The low levels of energy (15kV1) used in the surface treatment appeared to confer a polishing effect by eliminating scratches and surface defects not completely removed by the polishing techniques utilized in sample preparation, an observation particularly marked in the first set of samples.

Modification of surface topography can arise either as a reciprocal bi-product of material bulk modifying technique (i.e., ECAP), or as a consequence of a focused surface modifying process. Only the immediate surface of an orthopedic device comes into intimate contact with the bone, simplifying the biological, mechanical and economic considerations of the depth of surface modifications required for effective implant/bone interaction. Furthermore, this reduces concerns relating to the effect of these modifications on the bulk material properties of the implants. Thus, LAEB treatment was shown to modify only the outer 10 μm layer of the metal surface (Proskurovsky et al., 1998).

The ideal implant surface finishing technique should not only guarantee rapid attainment of long-lasting secondary stability with consequent positive impact on clinical outcomes, but, from a manufacturing perspective, should be cost-effective and reliable. The concept of cost-effectiveness covers all aspects that reduce the cost of manufacturing, including the speed and cost of the surface finishing technique, as well as the balance of benefits versus deficiencies conferred. LAEB treatment is rapid and able to cover large areas of non-planar geometry from a single electron beam gun. The ensuing potential benefits offered by this treatment technique of orthopedic implants include: (i) the induction of osteoinductive properties under discretely defined treatment parameters, (ii) hardening of the surface and, (iii) improvement of corrosion characteristics without affecting the bulk material properties (Gao, 2013; Walker et al., 2014). However, further innovation and refinement of uncemented implant technology will require tightly controlled and cautious post-market evaluation and review facilitated by experts, as endorsed by BOA and Beyond Compliance (BOA, 2013; BeyondCompliance, 2015). The industrial attraction of the LAEB process for large-scale production lies in the relatively low cost, high electrical efficiency, reliability, facile control, large beam diameter, and X-ray safety of the source (Rotshtein et al., 2006; Batrakov et al., 2008).

In summary, the current findings indicate that LAEB offers significant potential as a large-scale osteoinductive orthopedic implant finishing processes. However, further evidence of LAEB-stimulated enhancement of osseointegration will require data from *in vivo* studies, particularly addressing histological extracellular osteoid matrix organization and mineralization as an integral part of osteogenesis, and evidence of mechanical interface strength enhancement and these studies are on-going in our groups.

## Ethics statement

This study was carried out in accordance with the recommendations of and was approved by the local Southampton General Hospital ethics committee approval (LREC194/99/1). All subjects gave written informed consent in accordance with the Declaration of Helsinki.

## Author contributions

VG, RC, JW, JM, AC, and RO designed the experiments. VG performed the experiments with support from RC, JW, and JM for materials manufacture and characterization. VG with RC, DD, and RO analyzed the results. VG and RO wrote the paper. All authors approved and contributed to the final draft.

### Conflict of interest statement

The authors declare that the research was conducted in the absence of any commercial or financial relationships that could be construed as a potential conflict of interest.

## Supplementary material

Supplementary Figure 1 Schematic of the SODICK PF32A Large Area Electron Beam irradiation machine.

Supplementary Figure 2 Real time qPCR analysis of osteogenic (ALP, Collagen type I, OPN and OCN—**A**) and negative control (Collagen type II—**B**) gene expression in STRO 1 SSCs cultured *in vitro* for 21 days. Ti64 Untreated is taken as a negative control. Results expressed as mean ± SD, triplicate samples, individual experiment repeated three times, 2-way ANOVA test, ^*^*p* < 0.05.
