# Supplementary figures and images for: Human Skeletal Stem Cell Response to Multiscale Topography Induced by Large Area Electron Beam Irradiation Surface Treatment
Source: Front Bioeng Biotechnol. 2018 Jul 24;6:91. doi: 10.3389/fbioe.2018.00091 (PMC6066554; doi:10.3389/fbioe.2018.00091)

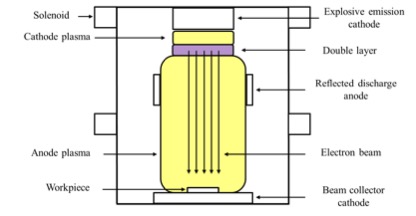

Supplement: Supplementary Figure 1 — Schematic of the SODICK PF32A Large Area Electron Beam irradiation machine. [file Image_1.JPEG]

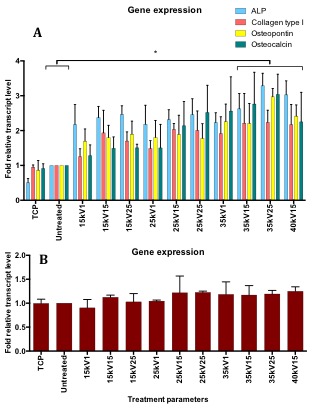

Supplement: Supplementary Figure 2 — Real time qPCR analysis of osteogenic (ALP, Collagen type I, OPN and OCN—A) and negative control (Collagen type II—B) gene expression in STRO 1 SSCs cultured in vitro for 21 days. Ti64 Untreated is taken as a negative control. Results expressed as mean ± SD, triplicate samples, individual experiment repeated three times, 2-way ANOVA test, *p < 0.05. [file Image_2.JPEG]
